# Predictive Factors for 30-Day Readmissions in Elderly Patients With Pneumonia: A Single-Center Retrospective Cohort Study

**DOI:** 10.7759/cureus.51380

**Published:** 2023-12-31

**Authors:** Yuuto Tonouchi, Yuki Kataoka

**Affiliations:** 1 Department of Rehabilitation, Kyoto Min-iren Asukai Hospital, Kyoto, JPN; 2 Department of Healthcare Epidemiology, School of Public Health, Kyoto University Graduate School of Medicine, Kyoto, JPN; 3 Department of Internal Medicine, Kyoto Min-iren Asukai Hospital, Kyoto, JPN; 4 Department of Systematic Reviewers, Systematic Review Workshop Peer Support Group, Osaka, JPN

**Keywords:** neutrophil-to-lymphocyte ratio, retrospective cohort study, prediction factor, readmissions, elderly pneumonia

## Abstract

Background

Pneumonia is a major concern among the elderly, with high readmission rates after hospitalization. These readmissions increase medical costs and reflect the quality of hospital care. This study aimed to explore the predictive factors associated with readmission within 30 days among elderly patients with pneumonia.

Methodology

This retrospective cohort study utilized the existing medical records. We included patients with pneumonia aged 75 and above who were discharged from a community hospital between April 2016 and March 2022. Patients who died during hospitalization or were transferred to other hospitals were excluded. Sex, age, length of hospital stay, Barthel Index (BI) at discharge, height, weight, body mass index, blood test findings, presence of tube feeding, Charlson Comorbidity Index, neutrophil-to-lymphocyte ratio (NLR), and Geriatric Nutritional Risk Index were used as predictive factors. The primary outcome was readmission within 30 days of discharge. A logistic regression analysis was performed.

Results

We included 337 patients: 50 (15%) in the readmission group and 287 (85%) in the control group. Univariate logistic regression analysis indicated low BI at discharge, and the odds ratio (OR) for readmission was 0.99 (95% confidence interval (CI) = 0.98-1.00). In patients with hemoglobin 10.0 g/dL or less, the OR for readmission was 2.18 (95% CI = 1.08-4.28). In patients with an NLR of 5 points or more, the OR for readmission was 2.64 (95% CI = 1.30 -5.24). In patients with aspartate transaminase of 38 U/L or more, the OR for readmission was 2.99 (95% CI = 1.07-7.68). Multivariate logistic regression revealed that an NLR of 5 points or more (adjusted OR = 2.42, 95% CI = 1.12-5.14) was correlated with readmission in elderly pneumonia patients.

Conclusions

In elderly patients with pneumonia, a high NLR at discharge may be a potential predictor of readmission within 30 days. This could be a new finding of our study. By sharing these findings during patient discharge conferences, there is potential to assist the medical team, patients, and caregivers in predicting unforeseen short-term readmissions. Further high-quality research is required to verify the reproducibility of these findings.

## Introduction

Pneumonia is an important public health problem in the elderly, and readmission rates after hospitalization for pneumonia remain high. In fact, approximately one in six patients after hospitalization for pneumonia are readmitted within 30 days [1]. Moreover, unexpected readmission within 30 days of discharge increases medical costs. These readmissions may also be indicators of the quality of hospital care [2]. Therefore, predicting readmission within 30 days in elderly patients with pneumonia is crucial for optimizing care, as it enables preventive care and effective allocation of resources. This study specifically targeted individuals aged ≥75 years. The study focuses on Japan, which has the highest level of aging population among advanced nations, with individuals aged ≥75 years constituting approximately 15.5% of the total population. This surpasses the population of individuals aged 65-74 years [3]. In previous studies, sex and comorbidities have been considered predictive factors [4]. In addition, some prediction models [5] have combined multiple predictors; however, these mainly target patients aged 18 and above or 65 and above. Reports specifically verifying the target population as 75 and above were not found within the reviewed literature, indicating a potential lack of evidence for this specific demographic.

Recently, prognostic factors related to nutrition and metabolism have received increasing attention. The Geriatric Nutritional Risk Index (GNRI) [6] has been reported as a predictor of 90-day readmission in elderly patients [7]. The neutrophil-to-lymphocyte ratio (NLR), calculated as a simple ratio of neutrophil count to lymphocyte count measured in peripheral blood, is considered a biomarker that connects the two faces of the immune system, namely, the natural immune response by neutrophils and the adaptive immune response by lymphocytes. It is regarded as an independent prognostic factor for mortality in several diseases [8]. NLR has been reported to be a predictor of 30-day readmission in patients with hepatic encephalopathy [9] and 90-day readmission in patients with community-acquired pneumonia [10]. However, previous studies have not investigated its predictive value among elderly patients with pneumonia. This study aimed to explore factors predicting readmission within 30 days in elderly patients with pneumonia. Specifically, we focused on predictors related to nutrition and metabolism.

## Materials and methods

Study design and ethical approval

This retrospective cohort study utilized the existing medical records. Specifically, we analyzed the medical records of elderly patients with pneumonia while referencing some of the predictive factors reported in previous research. This study aimed to explore the factors predicting readmission. The Ethics Committee of Kyoto Min-Iren Chuo Hospital approved the research protocol (ID:137).

Study participants

**Figure 1:**
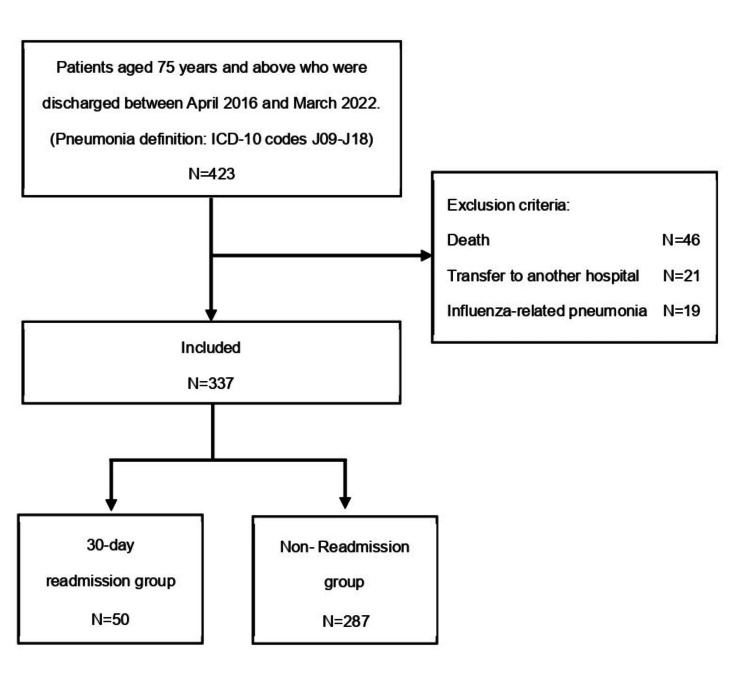
Study flowchart.

We included patients aged 75 or older who were admitted to our hospital with a diagnosis of pneumonia and discharged between April 2016 and March 2022. The definition of pneumonia includes individuals with the primary diagnosis code in the International Classification of Diseases 10th Revision (ICD-10) codes J09-J18 [11]. Patients with outcomes of death or transfer were excluded. Additionally, patients diagnosed with influenza (ICD-10 codes J09-J11) were excluded (Figure 1). COVID-19 pneumonia was not included as the hospital did not provide admission support for it.

Definition of predictors

The variables used for the survey were as follows: sex, age, length of hospital stay, Barthel Index at discharge used as an indicator to assess the level of independence in activities of daily living, height, weight, body mass index (BMI), blood test findings (white blood cell count (WBC), hemoglobin (Hb), platelet count (PLT), neutrophil count, lymphocyte count, albumin, sodium, creatinine, blood urea nitrogen (BUN), C-reactive protein (CRP), and aspartate aminotransferase (AST)), presence of nasogastric tube feeding, and Charlson Comorbidity Index (CCI). In addition, we calculated the neutrophil-to-lymphocyte ratio (NLR), an immune function biomarker, and GNRI, a nutritional risk assessment biomarker, from the above items and included them in the survey items (NLR = neutrophil count/lymphocyte count, GNRI = 14.89 × serum albumin + 41.7 × current weight in kg/ideal weight in kg). Data on sex, age, height, weight, and BMI were extracted from the discharge summary written by the physician. The most recent blood data obtained before the discharge date were extracted. The presence or absence of tube feeding was confirmed from the receipt data (medical act name “nasal nutrition”) one day before discharge. The CCI was calculated based on the ICD-10 codes of comorbidities in DPC data format 1 according to Quan’s algorithm [12].

BMI values were classified into the following three groups: underweight (<18.5 kg/m^2^), normal weight (18.5-25 kg/m^2^), and overweight (>25 kg.m^2^) [13]. The GNRI was classified into the following four groups: high nutritional risk (<82), moderate nutritional risk (82-92), mild nutritional risk (92-98), and no nutritional risk (>98) [6]. NLR was set at 5 for the cutoff value, consistent with the diagnostic criteria for sepsis, resulting in the classification of the following two groups: ≥5 points and <5 points [14]. The reason is that although previous studies have indicated several different cutoff values [10], all of them are 11 or higher, and in this study, using such cutoff values would result in a very small number of patients. Blood data were classified based on previous studies [15-17] as follows: WBC into the following three groups: <60 (×10²/µL), 60-120 (×10²/µL), and >120 (×10²/µL); Hb into the following two groups: <10.0 (g/dL) and ≥10.0 (g/dL), PLT into the following two groups: <36.0 (×10⁴/µL) and ≥36.0(×10⁴/µL); creatinine into the following two groups: <2.5 (mg/dL) and ≥2.5 (mg/dL), and BUN into the following two groups: <30 (mg/dL) and ≥30 (mg/dL). Sodium was classified into the following three groups: <135 (mmol/dL), 135-147 (mmol/dL), and >147 (mmol/dL) based on the hospital’s standard values because there were no corresponding cases when the standard value (<130 mmol/dL) reported in a previous study [16] was used. Other factors were also classified based on the hospital’s standard values: CRP into two groups as <0.3 (mg/dL) and ≥0.3 (mg/dL), albumin into two groups as <4.1 (g/dL) and ≥4.1 (g/dL), and AST into two groups as <38 (U/L) and ≥38 (U/L).

Definition of outcome

The primary outcome was the readmission to our hospital within 30 days of discharge. To validate the results, we randomly selected 30 patients without outcomes and manually reviewed electronic health records to confirm no readmissions within 30 days at other hospitals.

Statistical analysis

The patients were categorized into exposed and non-exposed groups for each of the aforementioned predictive factors, and the two groups were compared using univariate logistic regression analysis. In addition, we pre-specified prognostic factors from previous studies and clinical perspectives [18]. We conducted multivariate logistic regression analysis using these predictive factors. Data were presented as median (interquartile range (IQR)) or number (percentage), and the statistical significance level was set at 5%. The analysis was performed using the statistical software R Studio [19].

## Results

The analysis included 337 out of 423 cases. Patients with non-influenza viral pneumonia (ICD-10 code J12) were not identified during the patient extraction stage, so the final inclusion criteria were based on ICD-10 codes J13-18. The readmission group consisted of 50 (15%) individuals, while the control group comprised 287 (85%) individuals (Figure 1).

Table 1 presents the patient backgrounds. The sex ratio was almost equal, with 49% males and 51% females, and the median age was 87 years (IQR = 82-91). The median Barthel Index score at discharge was 22 points (median) in the readmission group and 50 points (median) in the non-readmission group. The NLR was 3.4 (median) in the readmission group and 2.9 (median) in the non-readmission group.

**Table 1:** Patient characteristics. ^1^: n (%); median (IQR). BMI: body mass index; WBC: white blood cell count; PLT: platelet count; BUN: blood urea nitrogen; CRP: C-reactive protein; AST: aspartate transaminase; CCI: Charlson Comorbidity Index; NLR: neutrophil-to-lymphocyte ratio; GNRI: Geriatric Nutritional Risk Index

	30-day readmission group, N = 50^1^	Non-readmission group, N = 287^1^	Overall, N = 337^1^
Gender			
Female	24 (48%)	148 (52%)	172 (51%)
Male	26 (52%)	139 (48%)	165 (49%)
Age	87 (82, 92)	87 (82, 91)	87 (82, 91)
Number of days in hospital	23 (16, 38)	22 (14, 42)	23 (15, 40)
Barthel Index (at discharge)	22 (0, 60)	50 (5, 85)	45 (5, 85)
Height (cm)	154 (145, 163)	153 (146, 160)	154 (146, 161)
(Missing)	0	4	4
Weight (kg)	44 (39, 52)	45 (39, 53)	45 (39, 53)
(Missing)	0	3	3
BMI (kg/m^2^)	19.2 (15.8, 22.8)	19.5 (16.9, 22.6)	19.5 (16.8, 22.6)
(Missing)	0	4	4
WBC (×10²/µL)	66 (53, 92)	58 (49, 72)	59 (50, 75)
(Missing)	4	43	47
Hemoglobin (g/dL)	10.50 (9.80, 11.40)	11.25 (10.20, 12.53)	11.20 (10.10, 12.40)
(Missing)	4	43	47
PLT (×10⁴/µL)	22 (19, 26)	23 (18, 29)	23 (18, 28)
(Missing)	4	44	48
Neutrophil count (×10^2^/μL)	43 (30, 73)	36 (29, 48)	37 (29, 50)
(Missing)	4	43	47
Lymphocyte count (×10^2^/μL)	13.9 (10.2, 17.4)	13.1 (9.6, 16.7)	13.2 (9.6, 16.8)
(Missing)	4	43	47
Albumin (g/dL)	3.00 (2.50, 3.20)	3.00 (2.70, 3.40)	3.00 (2.70, 3.30)
(Missing)	4	43	47
Sodium (mmol/dL)	139 (137, 141)	139 (137, 141)	139 (137, 141)
(Missing)	4	43	47
Creatinine (mg/dL)	0.80 (0.61, 1.00)	0.81 (0.63, 1.08)	0.81 (0.61, 1.07)
(Missing)	4	43	47
BUN (mg/dL)	19 (12, 27)	16 (12, 24)	17 (12, 24)
(Missing)	4	43	47
CRP (mg/dL)	1.19 (0.42, 2.41)	0.91 (0.24, 2.15)	0.98 (0.26, 2.25)
(Missing)	4	46	50
AST (U/L)	19 (15, 24)	19 (15, 23)	19 (15, 24)
(Missing)	5	45	50
Tube feeding	4 (8.0%)	7 (2.4%)	11 (3.3%)
CCI	3.00 (2.00, 4.00)	3.00 (2.00, 4.00)	3.00 (2.00, 4.00)
NLR	3.4 (1.9, 6.4)	2.9 (1.9, 4.2)	2.9 (1.9, 4.5)
(Missing)	4	43	47
GNRI	82 (71, 89)	82 (75, 90)	82 (74, 90)
(Missing)	4	46	50

The median CCI was 3.00 (IQR = 2.00-4.00) in both the readmission group and the non-readmission group. The median GNRI was 82 (IQR = 71-89) in the readmission group and 82 (IQR = 75-90) in the non-readmission group.

Table 2 shows the results of the univariate logistic regression analysis for each variable. The following results were obtained from the comparison between the readmission and non-readmission groups: in patients with low Barthel Index score at discharge, the odds ratio (OR) for readmission was 0.99 (95% confidence interval (CI) = 0.98-1.00). In patients with Hb of 10.0 g/dL or less, the OR for readmission was 2.18 (95% CI = 1.08-4.28). In patients with an NLR of 5 points or more, the OR for readmission was 2.64 (95% CI = 1.30-5.24). In patients with AST of 38U/L or more, the OR for readmission was 2.99 (95% CI = 1.07-7.68).

**Table 2:** Univariate analysis result. OR: odds ratio; CI: confidence interval; CCI: Charlson Comorbidity Index; BMI: body mass index; GNRI: Geriatric Nutritional Risk Index; NLR: neutrophil-to-lymphocyte ratio; WBC: white blood cell count; PLT; platelet count; BUN: blood urea nitrogen; CRP: C-reactive protein; AST: aspartate transaminase

	OR	P-value	95% CI	
Gender				
Female	[reference]		[reference]	
Male	1.15	0.64	0.63	2.11
Age	1.01	0.54	0.97	1.06
Number of days in hospital	1.00	0.53	0.98	1.01
Barthel Index (At discharge)	0.99	0.01	0.98	1.00
Height (cm)	1.00	0.94	0.97	1.03
Weight (kg)	0.99	0.69	0.96	1.02
Tube feeding	3.48	0.05	0.88	11.99
CCI	1.08	0.44	0.89	1.30
BMI (kg/m^2^)				
18.5-25	[reference]		[reference]	
<18.5	1.26	0.71	0.66	2.44
>25	1.49	0.41	0.54	3.70
GNRI				
>98	[reference]		[reference]	
<82	1.84	0.43	0.49	12.01
82–92	2.10	0.35	0.53	14.03
93–98	1.58	0.60	0.31	11.84
NLR				
<5	[reference]		[reference]	
≧5	2.64	0.01	1.30	5.24
WBC (×10²/μL)				
60–120	[reference]		[reference]	
<60	0.54	0.07	0.28	1.04
>120	1.09	0.90	0.23	3.81
Hemoglobin (g/dL)				
>10.0	[reference]		[reference]	
<10.0	2.18	0.02	1.08	4.28
PLT (×10⁴/μL)				
<35.0	[reference]		[reference]	
>35.0	0.90	0.81	0.40	2.31
Sodium (mmol/dL)				
135–147	[reference]		[reference]	
<135	0.94	0.90	0.34	2.25
>147	2.67	0.43	0.12	28.50
Creatinine (mg/dL)				
<2.5	[reference]		[reference]	
>2.5	0.67	0.59	0.10	2.41
BUN (mg/dL)				
<30	[reference]		[reference]	
>30	0.85	0.67	0.37	1.76
CRP (g/dL)				
<0.3	[reference]		[reference]	
>0.3	1.01	0.99	0.51	2.10
Albumin (g/dL)				
>4.1	[reference]		[reference]	
<4.1	0.94	0.96	0.15	18.26
AST (U/L)				
<38	[reference]		[reference]	
>38	2.99	0.03	1.07	7.68

Table 3 shows the results of the multivariate logistic regression analysis. Patients with an NLR of 5 points or more (adjusted OR = 2.42, 95% CI = 1.12-5.14) were correlated with readmission in elderly pneumonia patients.

**Table 3:** Multivariate analysis results. OR: odds ratio; CI: confidence interval; CCI: Charlson Comorbidity Index; NLR: neutrophil-to-lymphocyte ratio; AST: aspartate transaminase; GNRI: geriatric nutritional risk index

	Adjusted OR	P-value	95% CI	
Gender (male)	1.47	0.29	0.73	3.03
Barthel Index (at discharge)	0.99	0.03	0.98	1.00
CCI	1.01	0.93	0.81	1.26
NLR ≧5	2.42	0.02	1.12	5.14
Hemoglobin <10.0 g/dL	2.13	0.05	0.98	4.55
AST >38 U/L	2.43	0.10	0.80	6.86
GNRI <82	0.85	0.84	0.20	5.80
GNRI 82–92	1.27	0.77	0.30	8.80
GNRI 93–98	1.18	0.86	0.21	9.23

## Discussion

In this study, we conducted univariate and multivariate logistic regression analyses based on predictors extracted from electronic medical record data of 337 patients aged ≥75 years who were hospitalized at our hospital due to pneumonia. We found that a high NLR at discharge may predict readmission within 30 days in elderly patients with pneumonia.

The NLR may be a potential predictor of short-term readmission in elderly patients with pneumonia. Previous studies have reported that the NLR is a predictor of mortality [10,20] and three-month readmission in patients with community-acquired pneumonia [10]. However, to our knowledge, there are no reports on the NLR as an outcome of short-term (within 30 days) readmission in patients with pneumonia. This could be a new finding of our study. By sharing these findings during patient discharge conferences, there is potential to assist the medical team, patients, and caregivers in predicting unforeseen short-term readmissions.

The introduction of NLR at discharge into conventional prediction models for elderly patients with pneumonia may improve prediction accuracy. The NLR can be easily checked by anyone from the electronic medical record data, similar to the factors (sex, comorbidities) used in conventional prediction models, thus, minimizing the complexity. Further research is needed to compare and verify the prediction models with and without NLR.

In our study, apart from the NLR, we did not find any factors that could be predictors of short-term readmission in elderly patients with pneumonia, which is inconsistent with the results of previous studies. Several previous studies [4,15-17] have reported that factors such as male sex, laboratory findings (low Hb, high BUN, low sodium level, and high PLT count), and chronic comorbidities may be predictors of short-term readmission in patients with pneumonia. In addition, in a study [21] of 30-day mortality as an outcome of community-acquired pneumonia, factors such as age, laboratory findings (low albumin, high BUN), and comorbidities (chronic obstructive pulmonary disease, malignant tumor) were reported as predictors. One possible reason for the discrepancy in results is the difference in study patients between the previous studies and ours. Previous studies targeted patients admitted to tertiary emergency medical institutions or used administrative data submitted by these medical institutions. In our study, we targeted patients admitted to a single secondary emergency medical institution, and there may have been differences in patient backgrounds. In addition, many previous studies set the patients as ≥18 years or ≥65 years, and none of them were limited to ≥75 years, as in our study.

Our study has several limitations. First, it was a retrospective cohort study conducted at a single institution, and further studies are needed to verify the generalizability of our findings to other institutions, especially to super-aged individuals. Second, because this study used existing medical records, we conducted an exploratory examination based on predictors reported in previous studies owing to the problem of measurability. Therefore, we did not include all the factors mentioned in the conventional prediction model. Furthermore, it is noteworthy that more than 40 blood test results are missing in the dataset. This is a significant constraint that may impact the interpretation of the study results. Third, this study included all types of pneumonia excluding viral pneumonia. Additionally, we did not conduct a disease-specific examination for each ailment reported as a predictive factor in previous studies. Therefore, further research is needed to determine whether the setting of the patients could be a risk factor for readmission. Moreover, it is essential to note that this study was conducted during the COVID-19 pandemic, which might have influenced the characteristics of the study participants. Fourth, due to the insufficient sample size for the selected covariates, the model results may be unstable. To solve these problems, it is necessary to increase the sample size and conduct higher-quality research, such as multicenter prospective studies with predefined study participants and predictors.

## Conclusions

In elderly patients with pneumonia, a high NLR at discharge may be a potential predictor of readmission within 30 days. By sharing these findings during patient discharge conferences, there is potential to assist the medical team, patients, and caregivers in predicting unforeseen short-term readmissions. Further high-quality research is required to verify the reproducibility of these findings.
